# Reassessing rainfall in the Luquillo Mountains, Puerto Rico: Local and global ecohydrological implications

**DOI:** 10.1371/journal.pone.0180987

**Published:** 2017-07-07

**Authors:** Sheila F. Murphy, Robert F. Stallard, Martha A. Scholl, Grizelle González, Angel J. Torres-Sánchez

**Affiliations:** 1 U.S. Geological Survey, Boulder, Colorado, United States of America; 2 U.S. Geological Survey, Reston, Virginia, United States of America; 3 USDA-Forest Service, International Institute of Forestry, Río Piedras, Puerto Rico, United States of America; 4 U.S. Geological Survey, Guaynabo, Puerto Rico, United States of America; University of California San Diego, UNITED STATES

## Abstract

Mountains receive a greater proportion of precipitation than other environments, and thus make a disproportionate contribution to the world’s water supply. The Luquillo Mountains receive the highest rainfall on the island of Puerto Rico and serve as a critical source of water to surrounding communities. The area’s role as a long-term research site has generated numerous hydrological, ecological, and geological investigations that have been included in regional and global overviews that compare tropical forests to other ecosystems. Most of the forest- and watershed-wide estimates of precipitation (and evapotranspiration, as inferred by a water balance) have assumed that precipitation increases consistently with elevation. However, in this new analysis of all known current and historical rain gages in the region, we find that similar to other mountainous islands in the trade wind latitudes, leeward (western) watersheds in the Luquillo Mountains receive lower mean annual precipitation than windward (eastern) watersheds. Previous studies in the Luquillo Mountains have therefore overestimated precipitation in leeward watersheds by up to 40%. The Icacos watershed, however, despite being located at elevations 200–400 m below the tallest peaks and to the lee of the first major orographic barrier, receives some of the highest precipitation. Such lee-side enhancement has been observed in other island mountains of similar height and width, and may be caused by several mechanisms. Thus, the long-reported discrepancy of unrealistically low rates of evapotranspiration in the Icacos watershed is likely caused by previous underestimation of precipitation, perhaps by as much as 20%. Rainfall/runoff ratios in several previous studies suggested either runoff excess or runoff deficiency in Luquillo watersheds, but this analysis suggests that in fact they are similar to other tropical watersheds. Because the Luquillo Mountains often serve as a wet tropical archetype in global assessments of basic ecohydrological processes, these revised estimates are relevant to regional and global assessments of runoff efficiency, hydrologic effects of reforestation, geomorphic processes, and climate change.

## Introduction

Mountain areas receive a much greater proportion of the world’s precipitation than other environments, thus serving as “water towers” for nearby populations ([1], and references cited therein). Mountain ranges with a strong prevailing wind direction receive high precipitation on windward slopes as a result of adiabatic cooling of air masses and concomitant precipitation with topographic lifting, and lower precipitation on leeward slopes due to moisture removal and/or adiabatic warming with downwind or downslope airflow [2]. Mean annual precipitation in the Luquillo Mountains of eastern Puerto Rico, in contrast, has typically been mapped and/or described as having little to no difference in windward and leeward precipitation [3–9], with some exceptions [10–12]. Many researchers have observed an increase in rainfall with elevation (0–1,050 m) [8, 9, 13–15], and a simple polynomial equation correlating elevation and mean annual precipitation (MAP) [9] has generally been used to estimate MAP in Luquillo watersheds and, by application of a water balance, evapotranspiration (ET) [3, 9, 16, 17]. The Parameter-elevation Relationships on Independent Slopes Model (PRISM), a spatial model of island-wide MAP based on observed rainfall at long-term rain gages, elevation, topography, and distance from the ocean [6], has also been used to estimate MAP for modeling the effects of land use and climate change on water resources in eastern Puerto Rico (e.g., [18–21]). Although maps derived from PRISM show a marked windward/leeward precipitation gradient from the northeast to the southwest on the 8,870-km^2^ island of Puerto Rico, precipitation appears symmetric for windward and leeward slopes of the Luquillo Mountains [6].

**Fig 1:**
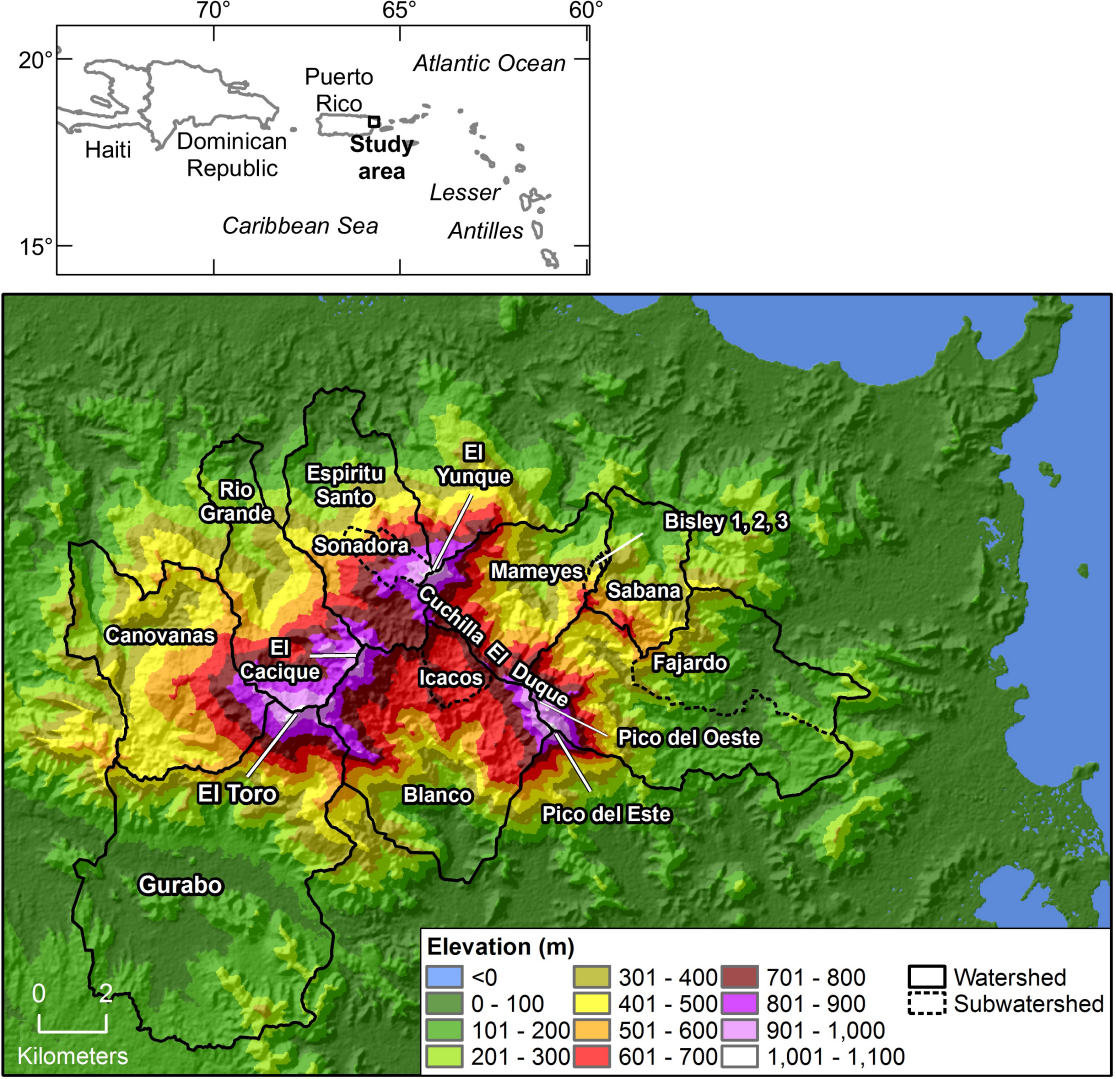
Map of Luquillo Mountains, Puerto Rico, showing elevation, topography, and watersheds discussed in text.

Discrepancies in several hydrological studies suggest that previous estimates of MAP in some regions of the Luquillo Mountains may have been inaccurate. Lower precipitation at rain gages on the western side of the Luquillo Mountains compared to similar elevations on the eastern side has been observed [12, 22, 23]. The Canóvanas watershed, which drains the western flank of the Luquillo Mountains (Fig 1), has about one-third the mean annual runoff of the east-draining Mameyes watershed, which is similar in size and elevation range [22]. Water-balance calculations for the Canóvanas watershed using MAP estimated by PRISM [19] required precipitation correction factors of 0.58 [22] and 0.721 [18] to close the water balance. Researchers have reported for decades that an unrealistically high proportion of precipitation exits as runoff from the Icacos watershed (with some reporting more runoff than rainfall); this finding has been attributed variously to underestimated rainfall, underestimated cloud water deposition, overestimated stream discharge, and/or high solar insolation [16, 22, 24, 25].

Water-budget-derived ET in Luquillo Mountain watersheds, when compared to ET determined by other methods in the region or to ET reported for other tropical forests, also suggests that watershed-scale estimation of MAP may not have captured spatial variability of rainfall. Recent global compilations have reported that while MAP in tropical forested watersheds ranges from 1,350–7,470 mm y^-1^, the range of ET is much smaller: generally between 800–2,000 mm y^-1^ [26, 27]. Forest ET increases, on a global average, about 46 mm for every 100 mm of rainfall [28]. Potential ET (PET) decreases with increasing rainfall, and in Puerto Rico PET and actual ET have been reported to be equal when MAP is >2,250 mm y^-1^ [29]. Potential ET (calculated with the Penman Monteith or Hargreave equations) and ET calculated by small-plot measurements of rainfall, throughfall, stemflow, and transpiration in the Luquillo Mountains have ranged from 1,010–1,560 mm y^-1^ [18, 30–33] (see review in [22], their Table 3). Water-budget-derived ET estimates for the Icacos watershed [9, 16, 22, 24, 34, 35] (and some for the neighboring Mameyes watershed [3]) are typically well below the range reported for tropical forests, while ET estimates for leeward watersheds have been above that range [3, 9]. Water-budget-derived ET in the small Bisley 2 watershed (Fig 1) was estimated to be ~2,300 mm y^-1^ (scaled up from sub-year time periods), about twice the ET estimated by a temperature fluctuation method and the Penman-Monteith equation [31]; this is the highest ET reported in global reviews of >800 forests [36, 37]. Canopy interception derived from those ET rates are about twice the rate estimated from subsequent direct interception measurements in the same forest [38], but have been included in assessments that suggest that maritime tropical forests have much higher rates of interception than continental tropical forests [39, 40]. A spatial analysis of the Luquillo Mountains that incorporated satellite imagery and a hydrologic model [17] (which estimated MAP with an elevation/MAP equation [9]), reported that ET was 0 to 89% of MAP (see Figure 2.3 in [17]); the low end of this range is physically unreasonable.

Errors in MAP and ET estimates for the Luquillo Mountains may have important ramifications for ecohydrological research, because the region has an outsized influence in comparisons of tropical and temperate processes. The El Yunque National Forest, which is conterminous with the Luquillo Experimental Forest (LEF) and is the only tropical rain forest in the U.S. national forest system, hosts the National Science Foundation-funded Luquillo Long-Term Ecological Research (LTER) program and Luquillo Critical Zone Observatory (CZO) program, along with the USGS Water, Energy, and Biogeochemical Budgets (WEBB) program. These programs have collectively hosted hundreds of scientific investigations (see, for example, reviews in [7, 13, 25, 41–43]), many of which have been incorporated into regional and global assessments of hydrology [19, 26, 27, 36, 44, 45], geochemistry [46–51], ecology [52], climate modeling [20, 21, 53–60], and social-ecological climate vulnerabilities [61–64]. A recent survey found that 62% of studies in two highly-rated tropical ecology journals were conducted in only ten countries; the sixth most important country for such studies was Puerto Rico [65]. The number of studies in tropical regions, in turn, is a small fraction of those in temperate regions [66]. The Bisley and Icacos watersheds often serve as prototypical very wet, tropical end members in geochemical, geomorphological, and hydrological studies; yet, as described earlier, discrepancies in water budgets for these watersheds have been unresolved.

Here we endeavor to revise and improve estimates of MAP and ET in the Luquillo Mountains. We hypothesize that the Luquillo Mountains, like many other mountain ranges around the world, have a substantial windward/leeward gradient, and that discrepancies in water budgets in the Luquillo Mountains are largely due to models of MAP that assume a consistent relation between elevation and MAP. We further hypothesize that rainfall/runoff relations and ET rates in the Luquillo Mountains, rather than being abnormally high or low, are similar to those of other tropical forests. In order to enhance spatially distributed estimates of rainfall, we 1) combine rainfall data from current and historical rain gages to obtain greater spatial diversity, over longer time intervals, than any previous study in the region; 2) examine several methods for spatial estimation of MAP, including satellite, radar, and geostatistical analysis of observed precipitation; 3) develop a revised map of MAP that shows a previously undescribed rain shadow in the Luquillo Mountains, and 4) use the revised map to estimate watershed-scale MAP. We compare the relation between estimated watershed MAP and mean annual runoff to previous studies in the Luquillo Mountains and other tropical and temperate regions. Finally, we address potential causative mechanisms for the spatial variation in rainfall, and identify gaps in spatial coverage and needs for future research.

## Methods

### Study area

The Luquillo Mountains rise steeply from sea level at the eastern coast of Puerto Rico to 1,050 m elevation in a distance less than 10 km (Fig 1). Five peaks in the range exceed 1,000 m elevation: El Yunque, Pico del Oeste, Pico del Este, El Cacique, and the highest, El Toro (1,075 m). These peaks, and the ridges connecting them, form a roughly horseshoe shape around an elevated plateau that opens to the south and is drained by the Río Blanco and its tributaries (including the Río Icacos). A ridge running from El Yunque peak to Pico del Este known as Cuchilla el Duque, with elevations between 750–1,050 m, serves as the western boundary of the Mameyes and Fajardo watersheds. The Sabana watershed originates at a ridge with a maximum elevation of 670 m, between the lower parts of the Mameyes and Fajardo watersheds. The Espíritu Santo and Río Grande watersheds comprise the area to the northwest of the horseshoe, while the Canóvanas and Gurabo watersheds drain the western and southwestern flanks of El Toro.

Although the occasional northern cold front induces precipitation in the Luquillo Mountains, the majority of precipitation is derived from tropical low-pressure systems, trade-wind orographic showers, and convective systems associated with easterly low-pressure waves transiting west across the Atlantic Ocean [67, 68]. Wind at the eastern coast of Puerto Rico is most frequently from the east [69, 70], and thus in this paper we consider the eastern Mameyes, Fajardo, and Sabana watersheds to be windward and the western Gurabo and Canóvanas watersheds to be leeward. The northern Espíritu Santo and Río Grande watersheds and the southern Blanco watershed are less easily placed in a windward/leeward context (as demonstrated later in this paper).

### Precipitation

Rainfall data from all available sources (networks operated by the National Oceanic and Atmospheric Administration [NOAA], U.S. Forest Service, and Luquillo LTER, along with shorter-term published studies) in and near the Luquillo Mountains were combined to obtain greater spatial diversity than in previous studies. Although some gages have operated for over a century, other studies lasted less than a year. For some gages, daily rainfall data were available, allowing for error identification and correction, whereas for others, only MAP for a certain number of years was reported. For existing or recent rain gages, exact location could be confirmed with a geographic positioning system (GPS); for historical rain gages, more extensive research was required to determine the location of the gage. Locations, periods of record, type of gage, and other details for each gage are provided in S1 Table. For computational purposes, rain gages were grouped by watershed. If a rain gage was located within 25 m lateral distance of another watershed, it was included in both watershed groups.

**Fig 2:**
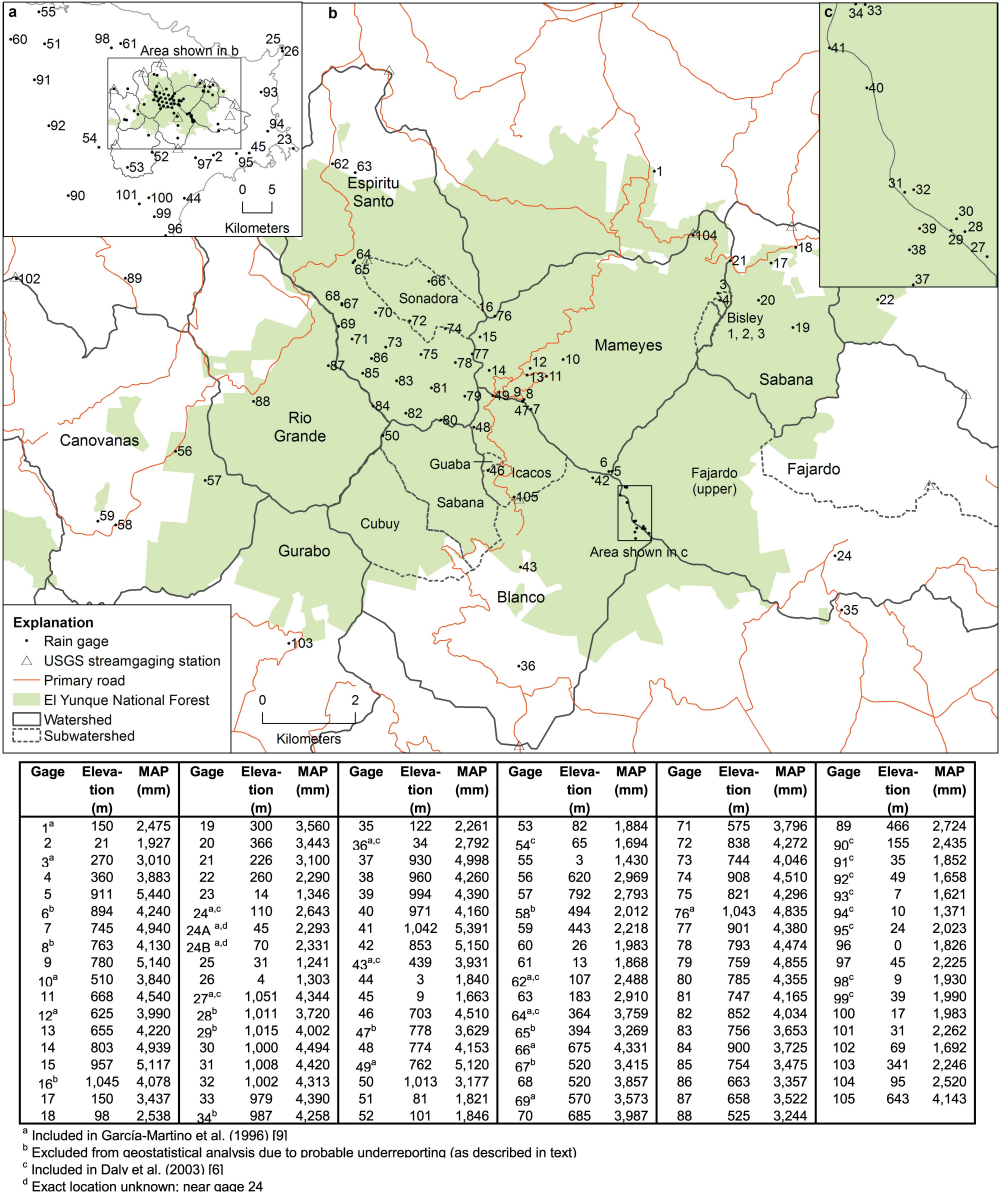
Map of rain gages (past and present) in the Luquillo Mountains, Puerto Rico, and table providing elevation and mean annual precipitation (MAP) for each gage (MAP for some gages were adjusted for low or high precipitation during short periods of record; see S1 Table for more details).

To assess interannual precipitation variability, and thus the effect of including rain gages with short periods of record, we analyzed the records of six long-term rain gages in eastern Puerto Rico (gages 27, 36, 43, 62, 93, and 98 in Fig 2; S1 Table). None of these gages had any long-term trend in annual precipitation (as determined with a Mann-Kendall test at p = 0.05 [71]). A minimum of nine years of precipitation data was required to result in a <10% difference between MAP for those years and mean MAP calculated for the entire period of record. Therefore, to estimate MAP for this study, if a station had nine or more years of record, we used the mean of that station as reported or calculated. If the station had less than nine years of record, we used the six long-term rain gages to compare the MAP for that time period to the long-term MAP. If that time period was unusually wet or dry, measured MAP was multiplied by the ratio of MAP for the period of record to that for time period at the nearest long-term gage (S1 Table).

**Fig 3:**
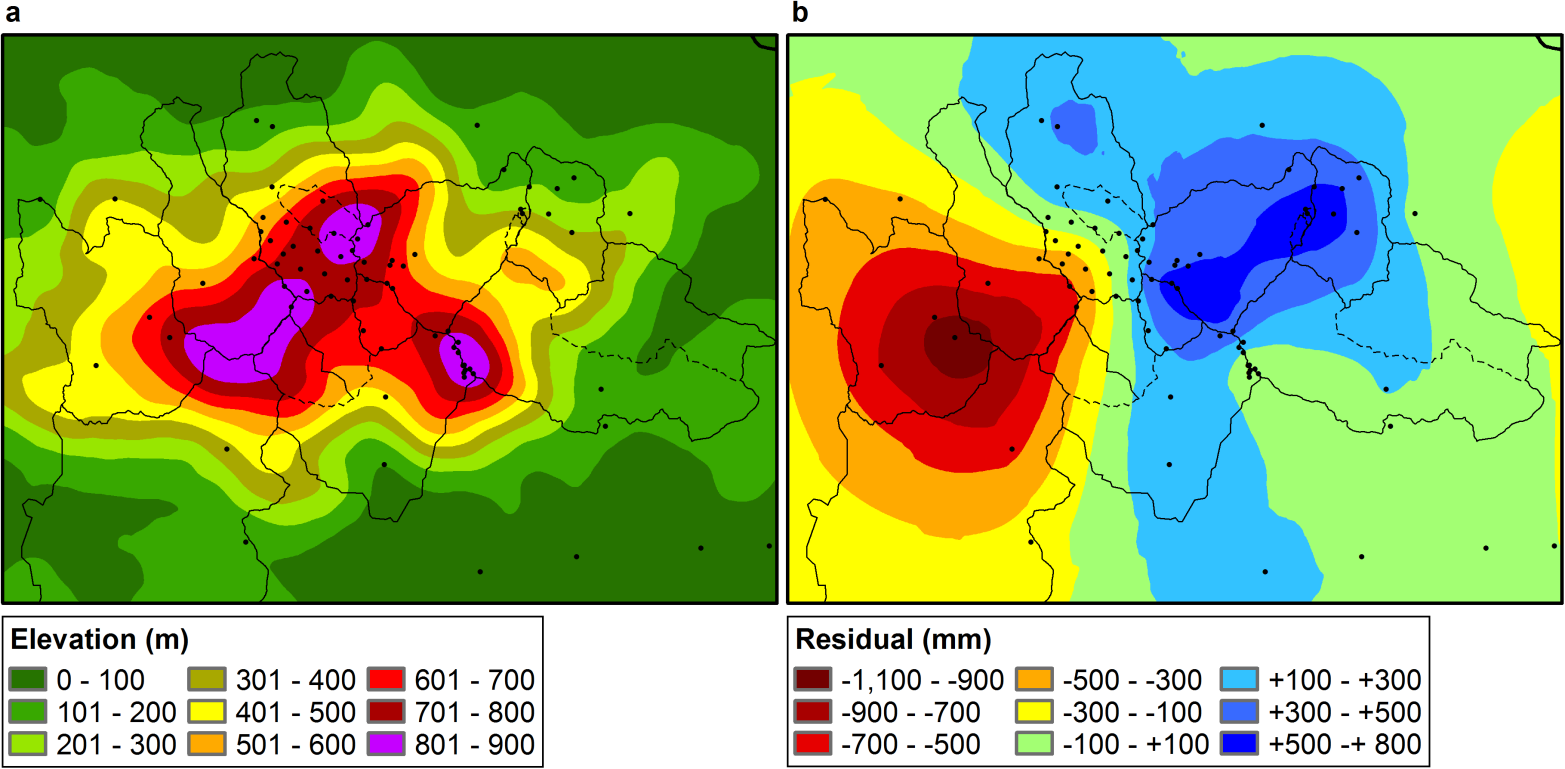
Maps of a) elevation filtered to a 1-km diameter and b) residual fields of mean annual precipitation interpolated with simple kriging using 24 neighboring stations.

We developed a spatial model of MAP for the Luquillo Mountains using elevation regression functions and residual interpolation, following the method of [72]. Previous studies have shown that precipitation patterns are more highly correlated with broad-scale topographic features than with local features [72–77]. Therefore, in order to determine the optimal scale for regression calculations, a focal averaging tool in a geographic information system (GIS) was used to filter a 10-m digital elevation model (DEM) [78] to mean elevations within circular neighborhoods (or “wavelengths”) of 0.1, 0.5, 1, 1.5, 2, 5, and 7 km diameter (S1 Fig). We then extracted the filtered elevations for each rain gage, and fit linear regressions between elevation (or wavelength) and measured MAP at rain gages in eastern Puerto Rico (excluding gages that we suspect grossly underreport MAP; Fig 2, S1 Table). The r^2^ value for the regression fitting reported elevation and MAP was 0.786, and slightly improved up to a 2-km wavelength (r^2^ 0.814) before declining. While the 2-km wavelength has the highest r^2^ value, filtering of elevation to this diameter results in substantial loss of topographic detail and no average elevations >800 m (S1 Fig). We therefore selected the 1-km wavelength (which retains key topographic features and results in an r^2^ value of 0.802) (Fig 3A, S1 Fig) to predict MAP at each gage, and calculated residuals (MAP_observed_−MAP_predicted_) for each gage. We then applied simple kriging to the residuals [79] with GIS (Fig 3B), developed a spatial model based on the equation MAP = 3.2117*1-km wavelength elevation + 1,843 + kriged residuals, and calculated MAP for each 10-m x 10-m cell. We then determined mean MAP for each watershed in the Luquillo Mountains.

Spatial estimates of MAP were also obtained from other available datasets, including PRISM [6] and radar- and satellite-derived rainfall data. The PRISM estimates are based on the period 1963–95 (PRISM has not since been updated for Puerto Rico). Modeling of measured MAP with PRISM used elevation as the predictor grid. The PRISM DEM, which had a 250-m grid resolution, was low-pass filtered to a 4.5-km effective wavelength (while maintaining the original 250-m resolution [6]). We downloaded radar-estimated MAP for Puerto Rico [80], which combines ground rainfall data and radar data (from a station located 30–40 km southwest of the Luquillo Mountains) and which is displayed as a gridded field with a spatial resolution of roughly 4.7 x 4.7 km. We converted these point data to a raster file and averaged radar-derived MAP for all available years (2005–2014, excluding 2013, which was not available). Both PRISM and radar-derived MAP were resampled to a 10-m x 10-m cell to match the resolution of our spatial model. We also evaluated the use of rainfall estimated from the Tropical Rainfall Measuring Mission (TRMM; [81]), which provides estimated rainfall at a grid size of 0.25 x 0.25 degrees. This grid size, which is about 28 km x 26 km in Puerto Rico, is too large to be useful in this study, and we did not pursue TRMM data further.

As an alternative approach to the spatial model, we developed polynomial equations for the relation between elevation and MAP for rain gages in the Luquillo Mountains following the method of [9]. We also developed separate polynomial equations for windward (eastern), leeward (western), northern, and southern watersheds. These equations exclude gages that we suspect underrecord precipitation (S1 Table; discussed further in Results). The fraction of each watershed within 50-m contour intervals was determined using the 10-m DEM [78], and that fraction was multiplied by the average MAP estimated at the midpoint of each 50-m ground-elevation interval using the relevant equation. We then summed these values to estimate MAP for each watershed.

For comparison of topography and precipitation in the Luquillo Mountains to other tropical islands in the trade wind region, we searched the literature for sites with spatially dense studies of precipitation. We acquired geospatial maps of elevation and MAP for the Hawaiian Islands of Maui and Oahu [82, 83]. Also, we obtained elevation data for the Indian Ocean island of Réunion [84] and digitized and interpolated isopleths [85]. Similarly, for the Pacific Island of New Caledonia, elevation data were obtained [78] and isopleths [86] were digitized and interpolated. We then used GIS to develop transects of elevation and MAP versus distance from the ocean from the dominant wind direction, which was from the northeast for Maui, Oahu, and New Caledonia, and from the east for Réunion. Transects began at x 799798, y 2306425 (Maui), x 615154, y 2387374 (Oahu), x 185528, y 52288 (Réunion), and x 276132, y 404626 (New Caledonia).

### Stream runoff

Mean annual discharge at USGS stream gages [87] was converted to annual runoff by dividing annual mean stream discharge by watershed area. Annual runoff was averaged to obtain mean annual runoff. We divided Luquillo watersheds into reference (little or no water management upstream) and non-reference watersheds based on the USGS GAGES-II dataset [88].

## Results

We compiled MAP data and geographic locations for over 100 rain gages in the Luquillo Mountains and surrounding area, spanning more than 100 years (Fig 2, S1 Table). Not surprisingly, gages in the Luquillo Mountains were almost always located near roads and (less frequently) trails, in areas where rainfall data were needed for water supply, flood warning, agriculture, transportation planning, or scientific studies. Many more gages were located in the windward watersheds than in leeward watersheds. Large gaps of spatial coverage, including a ~45-km^2^ contiguous area in the headwaters of the Canóvanas, Gurabo, Río Grande, and western Blanco watersheds, and a similar-sized area in the Mameyes, Fajardo, and Sabana watersheds, reflect the difficulty in accessing much of the region. While we identified 14 gages in the Fajardo watershed, none were between 150–900 m elevation, which represents 65% of the watershed area. Spatial distribution of gages in the Espíritu Santo watershed was dense due to some short-term studies, but nearly all gages in the Blanco watershed were located along roads in the eastern half (Fig 2).

This merging of datasets and individual studies revealed that substantially different precipitation amounts have been recorded at rain gages in the Luquillo Mountains that had been located within meters of each other. Such differences were greatest if one gage was on a tower and the other was close to the ground. Gage 65, on a 20-m tower had 13% less MAP than gage 64, located nearby on a building rooftop (Fig 2). Gages located on shorter towers (3 m), however, often had *higher* MAP than nearby gages closer to the ground. Different types of gages were used for different time periods (S1 Table), and thus we cannot compare gage type or make corrections (e.g. [89]). Rainfall measurement errors (such as gage malfunction, wind-field distortion, and blocking by vegetation) and the omission of horizontal precipitation and cloud-water deposition typically result in lower precipitation totals than “true” precipitation [90, 91]; therefore, when gages that had operated in the same location were substantially different, the gage with lower MAP was excluded from further analysis (Fig 2, S1 Table).

**Fig 4:**
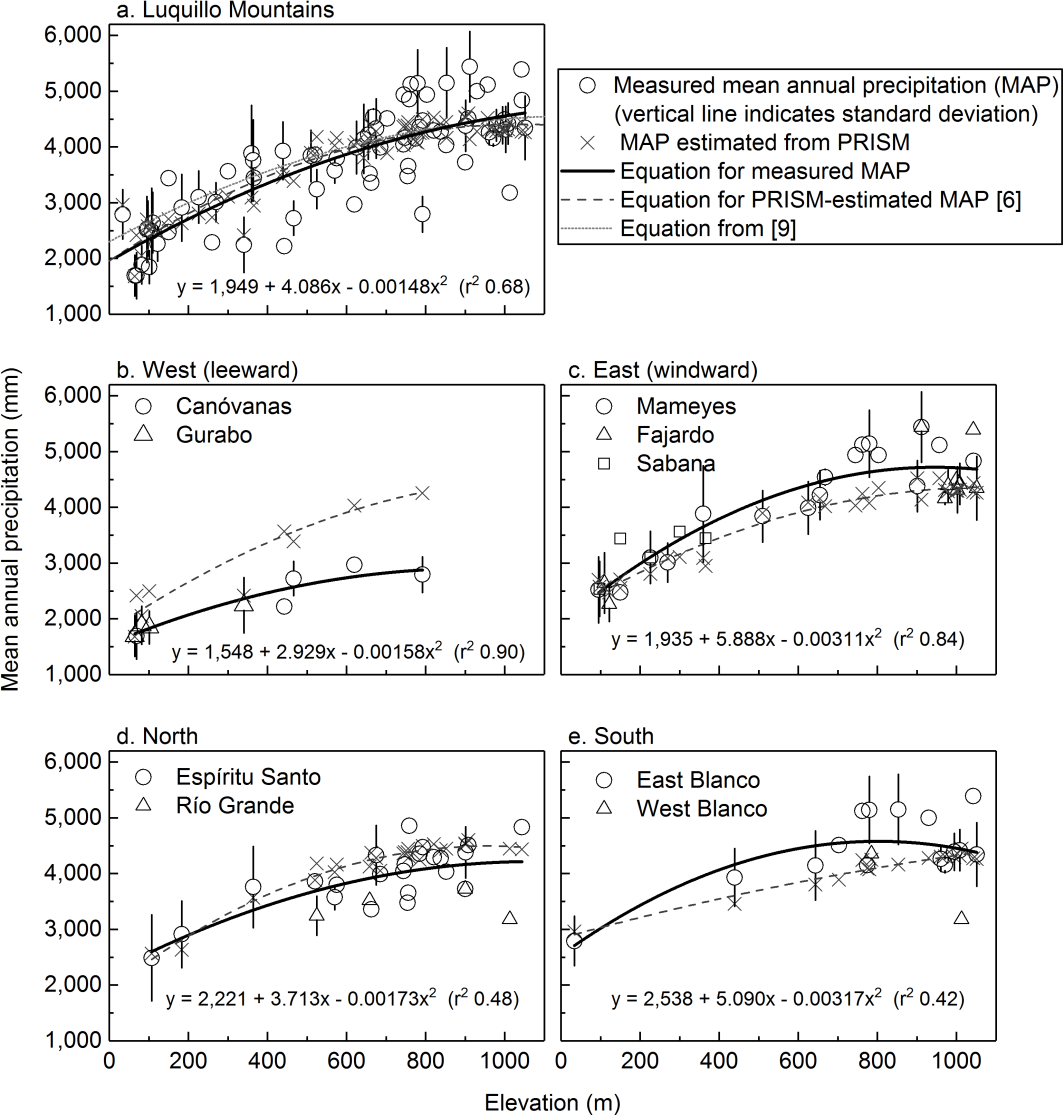
Elevation versus mean annual precipitation measured at rain gages and as predicted for those gages by the Parameter-elevation Relationships on Independent Slopes Model (PRISM [6]) in the Luquillo Mountains for a) all watersheds (polynomial equation from [9] also shown); b) western (leeward); c) eastern (windward); d) northern; and e) southern watersheds.

The plot of elevation versus MAP in the Luquillo Mountains for this new compilation (excluding gages that we suspect underreport; S1 Table) shows a general increase in precipitation with elevation (Fig 4A), but a polynomial equation has a much weaker correlation (r^2^ = 0.68) than reported earlier (r^2^ = 0.91) [9] or obtained if MAP at those locations is estimated with PRISM (r^2^ = 0.92) [6]. Thus, while elevation plays a large role in determining MAP in the Luquillo Mountains, other factors must also be involved.

**Fig 5:**
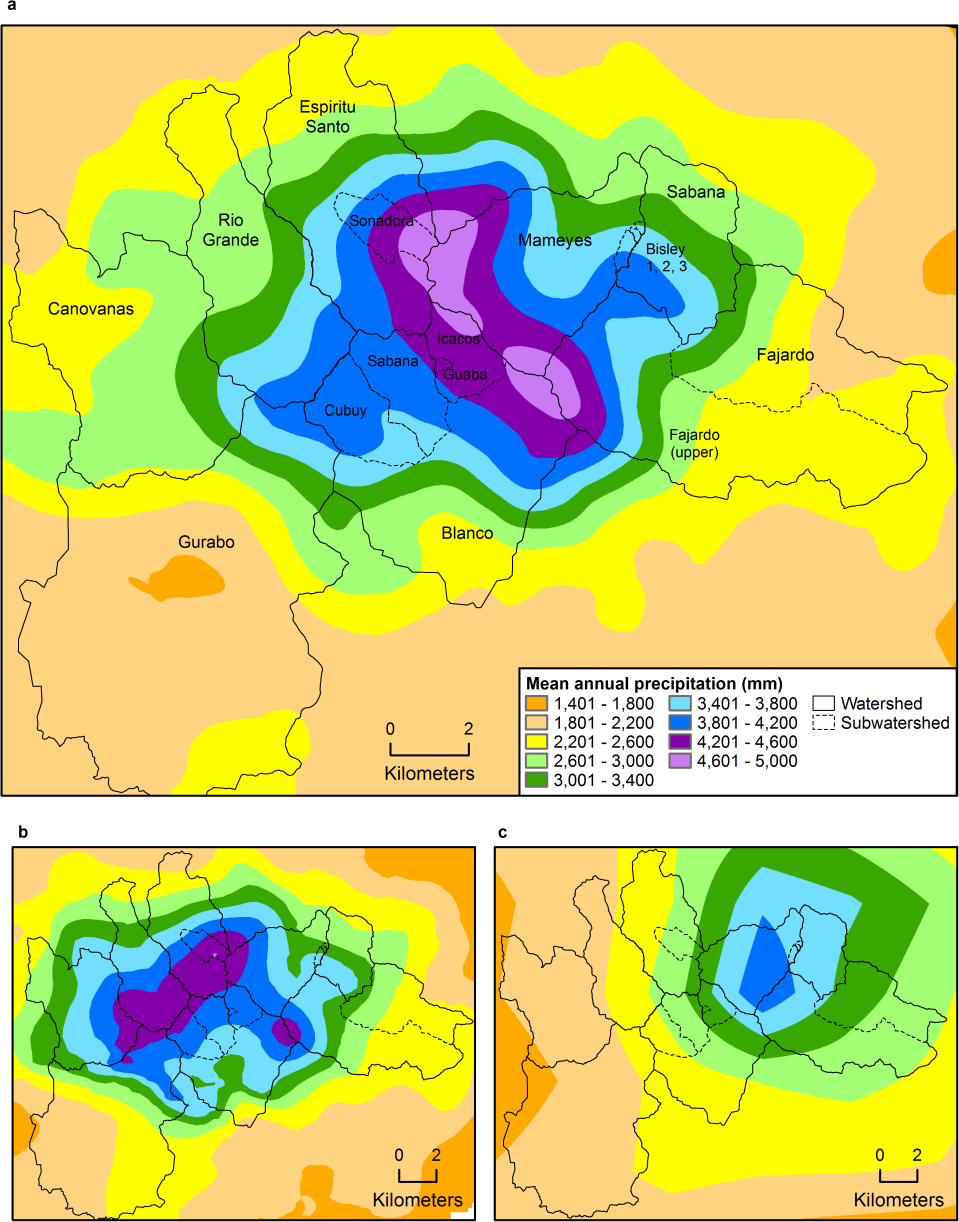
Maps of Luquillo Mountains, Puerto Rico, showing mean annual precipitation as predicted by a) spatial model developed from 1-km wavelength/MAP regression and residual interpolation; b) PRISM [6]; and c) radar [80].

Both the spatial model and the elevation/MAP equations for this new compilation show that western (leeward) watersheds of the Luquillo Mountains receive substantially lower MAP than eastern (windward) watersheds (Figs 4 and 5A, S2 Fig). Individual rain gages on western slopes received up to 46% less MAP than those at equivalent elevations on eastern slopes; this difference was greater at higher (>300 m) elevations than lower (<150 m) elevations (Fig 4). The leeward Canóvanas watershed receives about a third less MAP than the eastern Mameyes watershed, despite being similar in size and mean elevation (Table 1). The spatial model shows that highest MAP (4,600–5,000 mm y^-1^) occurs in a band along Cuchilla el Duque, the ridge separating the Mameyes and Fajardo watersheds from the Espíritu Santo and eastern Blanco watersheds (Figs 1, 5A and 5B). Mean annual precipitation immediately to the west (lee) of that ridge, in both the Espíritu Santo and Blanco watersheds, was similar to or higher than that in the windward watersheds at equivalent elevations. Precipitation then decreases to the west; MAP for the Río Grande watershed was between that of windward and leeward watersheds. The rainfall gradient may be relatively sharp: annual rainfall decreased by ~15% in two 780-m northeast-southeast transects across the main channel of the Espíritu Santo [92]. Discerning spatial variability of MAP in the southern Blanco watershed is more challenging; due to the lack of gages in the western half (Fig 2), there is substantial uncertainty in any precipitation map or watershed-wide MAP estimates in this region.

**Table 1:** Characteristics of watersheds in the Luquillo Mountains, Puerto Rico, and estimates of mean annual precipitation, runoff, and annual evapotranspiration.

Watershed		USGS stream gage	Area (km^2^) ^a^	Mean eleva-tion (m) ^b^	Mean annual precipitation (mm)							P—R ^i^			
					Spatial model ^c^	Equa-tion ^d^	PRISM ^e^	Radar ^f^	Regress-ion of tropical forests ^g^	Literature	Mean annual runoff (mm) ^h^	Spatial model	Tropical regress-ion	Annual evapotrans-piration (mm) from literature	Literature sources
**East**															
Mameyes		50065500 ^j^	17.8	505	3,953	3,989	3,649	3,715	4,297	3,570–4,235	2,856	1,097	1,441	725–1,904	[3, 9, 16, 22]
	Bisley I	—	0.062	350	3,417	3,609	2,968	3,683	—	3,596–3,630	—	—	—	—	[45, 93, 94]
	Bisley II	—	0.064	363	3,539	3,657	3,094	3,701	—	3,584–3,630	—	—	—	1,039–2,420	[17, 31, 45, 93, 94]
	Bisley III	—	0.330	476	3,770	4,009	3,453	3,753	—	—	—	—	—	—	[45]
Sabana		50067000	10.3	325	3,269	3,448	3,026	3,541	3,210	3,220–3,329	1,747	1,522	1,463	1,378–1,586	[3, 9]
Fajardo		50071000	37.0	274	2,824	3,170	2,859	3,102	3,022	2,875–3,430	1,555	1,269	1,467	1,449–1,838	[3, 9, 17, 18]
	Fajardo	50070900 ^j^	24.5	330	3,014	3,354	3,013	3,144	3,088	—	1,622	1,392	1,466	—	—
**South**															
Blanco		50076000 ^j^^,^^k^	31.9	527	3,631	4,140	3,683	2,776	4,010	3,730	2,563	1,068	1,447	2,052	[3]
	Icacos	50075000 ^j^	3.26	683	4,447	4,528	3,973	3,300	5,313	4,153–4,310	3,893	554	1,420	306–1,208	[3, 9, 16, 22, 24, 34, 35]
	Guabá	50074950 ^j^^,^^k^	0.11	704	4,283	4,547	3,913	3,151	4,820	—	3,390	893	1,430	—	—
	Sabana	—	4.54	—	4,005	—	3,932	2,857	—	—	—	—	—	—	—
	Cubuy	—	4.46	—	3,872	—	3,979	2,561	—	—	—	—	—	—	—
**West**															
Gurabo		50055750	57.8	215	2,216	2,044	2,581	1,995	2,130	3,600	645	1,571	1,485	2,860	[3]
Canóvanas		50061800	25.5	463	2,680	2,523	3,510	1,912	2,474	1,722–3,600	996	1,684	1,478	930–2,630	[3, 16, 18, 22]
**North**															
Río Grande		50064200	18.9	517	3,109	3,565	3,762	2,253	3,542	3,732	2,086	1,023	1,456	1,529	[9]
Espíritu Santo		50063800	22.3	459	3,442	3,420	3,529	2,616	3,874	3,743–3,750	2,424	1,018	1,450	1,339–1,434	[3, 9]
	Sonadora	50063440 ^j^^,^^k^	2.6	740	4,298	3,981	4,324	2,789	4,249	4,203–4,377	2,807	1,491	1,442	1,822	[9, 34]

^a^ From USGS [87] except Bisley [94], Guabá [22], and Blanco subwatersheds Cubuy and Sabana (derived in geographic information system (GIS) from 10-m elevation [78]).

^b^ Weighted mean area elevation (determined with GIS from 10-m elevation [78]).

^c^ From spatial model shown in Fig 5A.

^d^ Elevation/mean annual precipitation equation from Fig 4.

^e^ Parameter-elevation Relationships on Independent Slopes Model (Fig 5B) [6].

^f^ Radar-derived MAP (Fig 5C) [80].

^g^ Calculated from runoff using regression from [27]; due to diversions, this is a minimum value for non-reference watersheds.

^h^ Mean annual runoff (mean annual discharge at USGS streamgages [87] divided by watershed area) for period of record (except Icacos, which excludes 1947–1952 due to much higher discharge and lack of information about gage during this period, and Sonadora and Guabá, which exclude 1994–1999 and 2003–2012, respectively, due to statistical anomalies in the published discharge records computed for these periods).

^i^ Does not account for storage, diversions, septic or wastewater inputs, or cloudwater deposition.

^j^ Reference watershed (no or very little water management upstream) [88].

^k^ Discontinued streamgage.

## Discussion

### Comparison to previous studies

Watershed estimates of MAP (determined with the spatial model shown in Fig 5A) indicate that while the windward Mameyes, Fajardo, and Sabana watersheds receive similar MAP to that estimated in previous studies, the leeward Canóvanas and Gurabo watersheds receive 20–40% less MAP (Fig 5A, Table 1). This difference is primarily due to the lower spatial density and restricted locations of rain gages used in previous studies, which largely reflect the location of long-term ecological studies (e.g., the El Verde Field Station, gage 64; Pico del Este, gage 27; and the Bisley watersheds, gages 3 and 4; Fig 2). A previous elevation/MAP equation incorporated 18 gages in the Luquillo Mountains [9], but none were located in the leeward Gurabo or Canóvanas watersheds (Fig 2). Another limitation pointed out by authors of that study was the lack of gages between 716 and 1,030 m elevation. Compared to PRISM [6], our MAP estimates for all windward watersheds except Fajardo are 8–15% higher, and our estimates for the leeward watersheds and the Río Grande watershed are 14–24% lower (Table 1). The PRISM modeling dataset [6] included six rain gages in the Luquillo Mountains (Fig 2), but none of these gages were on the leeward slopes; the nearest leeward gage available to PRISM was located in a valley to the southwest of the Luquillo Mountains (gage 54 in Fig 2). While PRISM has the ability to discern windward/leeward exposures to capture rain shadow effects, sufficient station data must be available to represent these exposures. In this case, a lack of such data resulted in smooth isopleths determined primarily by elevation, with little windward/leeward difference (Fig 5B). The spatial model of MAP presented here results in higher MAP for the Icacos watershed than does PRISM due to the inclusion of 11 gages that recorded 4,600–5,500 mm y^-1^ on or within 300 m of the ridge line of Cuchilla el Duque (Figs 1, 2 and 5A), where PRISM predicted a maximum of 4,600 mm (Fig 5B). We also note that long-term rain gages in eastern Puerto Rico received 2–7% less MAP during the period modeled by PRISM (1963–1995) than during the entire period of record. Our elevation/measured MAP equations are closest to PRISM estimates at <150 m elevations in the Mameyes, Fajardo, and eastern Blanco watersheds, and at middle elevations in the Espíritu Santo watershed (Fig 4), because PRISM included gages at those elevations.

Radar-derived MAP shows a windward/leeward difference in the Luquillo Mountains (Fig 5C), but underestimates MAP substantially in most regions. This underestimation was also observed in a coarser study of the entire island of Puerto Rico [95]. Similar to findings in western Puerto Rico [96], radar does not effectively capture the spatial variability of MAP in eastern Puerto Rico. Radar-derived MAP was closest to measured MAP in the Gurabo and Canóvanas watersheds (Table 1), which are closest and most similar in elevation to the radar station. In all other Luquillo Mountain watersheds, mountains obstruct radar from reaching lower altitudes in the atmospheric column, which decreases radar-derived MAP. Other issues with radar-based precipitation estimates include sampling errors caused by freezing or frozen precipitation (possible here at >5 km altitude), bright banding, accuracy of the reflectivity/rainfall relationship in use, and radar calibration [80].

**Fig 6:**
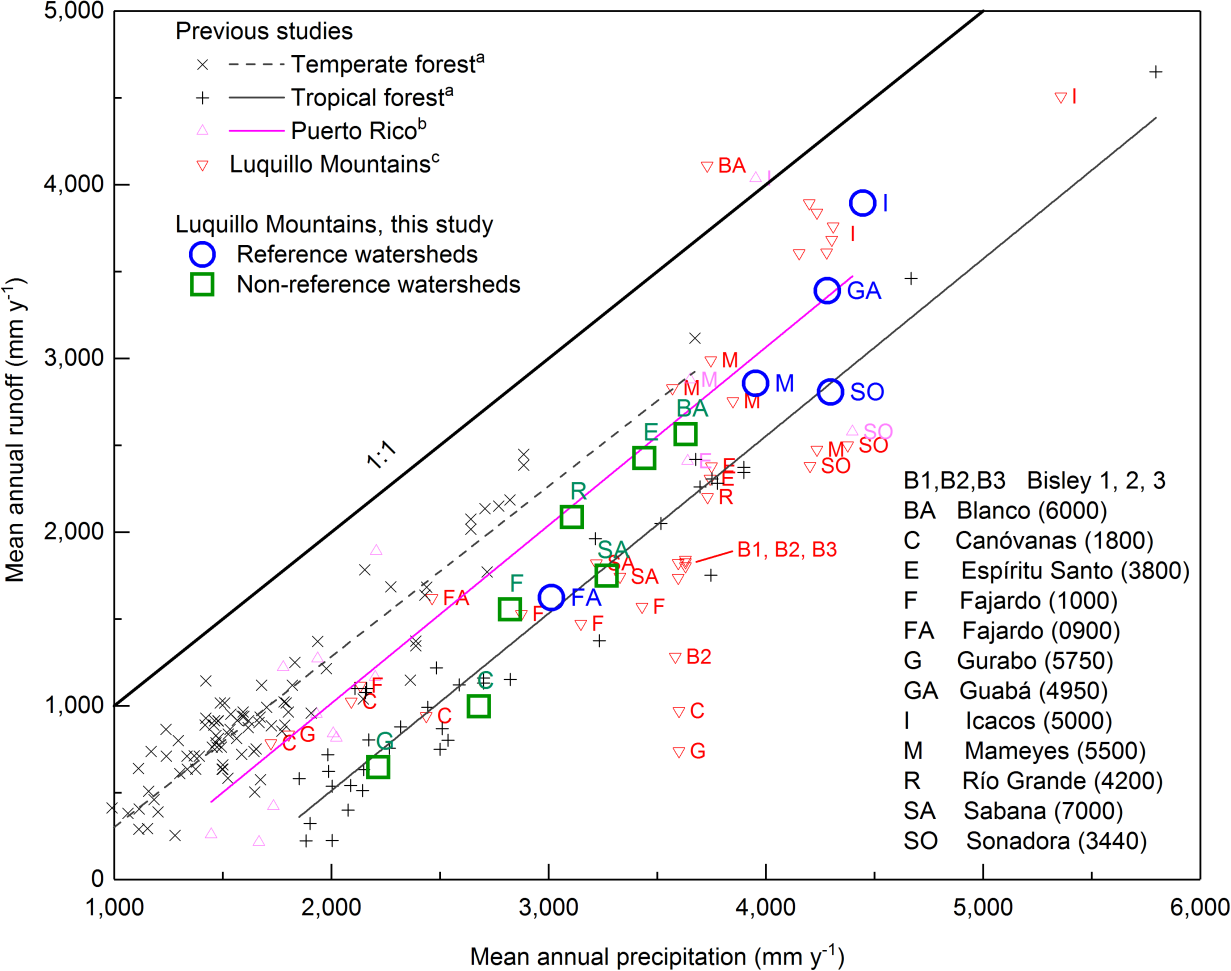
Mean annual precipitation versus mean annual runoff for temperate and tropical forests, Puerto Rico, and Luquillo Mountains (previous studies: ^a^ [27], ^b^ [19], ^c^ [3, 9, 18, 22, 24, 34, 35, 45, 103, 104]).

Linear relations between MAP and mean annual runoff have been observed in temperate forests and in tropical forests across the globe [27], with relatively less runoff from tropical forests than temperate forests due to higher ET. When we compare watershed estimates of MAP derived from the spatial model (Fig 5A) to mean annual runoff from USGS-gaged watersheds (Table 1), the watersheds generally fall along or near the mean relation for tropical forests (Fig 6). In contrast, a compilation for Puerto Rico [19], which used PRISM [6] to estimate MAP and USGS data [88] for mean annual runoff in reference watersheds (those with no or very minor water management upstream), indicates a linear relation that lies closer to temperate forests than to tropical forests. In previous Luquillo-specific water-budget studies, MAP versus mean annual runoff varied widely (Fig 6); the leeward Gurabo and Canóvanas watersheds and the small Bisley and Sonadora subwatersheds often plotted to the right (indicating runoff deficiency) of the mean relation for tropical forests while the Mameyes and Blanco watersheds (including the Icacos subwatershed plotted well to the left (runoff excess).

Although MAP estimates determined from the spatial model result in many Luquillo watersheds plotting closer to the tropical trend line than previous studies [3, 9, 16, 22, 24, 35], several still appear to have runoff excess forest trend line (Fig 6). We suspect that currently available precipitation data still leads to underestimated MAP, particularly in the Icacos watershed. If mean annual runoff from the Icacos watershed is accurate, and if this watershed has a similar rainfall/runoff relation as other tropical forests [27], then MAP could be as much as 5,313 mm y^-1^, which is 22% higher than the spatial model (Fig 5A) would indicate (Table 1). While measured MAP at gage 105, near the mouth of the Icacos watershed, was 4,143 mm (Fig 2), higher than most previous estimates of MAP for the watershed (Table 1), it is a tipping-bucket rain gage and is thus vulnerable to underrecording of both high-frequency, low-intensity rainfall and low-frequency, high-intensity rainfall. Studies from southern Ecuador found that such gages underrecord MAP by 15% [89, 97]. Also, the gage is elevated (located on the roof of a shelter), which can distort the wind field, causing rain drops to be carried over the orifice and resulting in up to 15% undercatch [90]. We note that annual rainfall and runoff in the Icacos watershed were estimated to be 5,359 mm y^-1^ and 4,572 mm y^-1^, respectively, in two publications from the 1960s [98, 99]; however, these papers cited unpublished Puerto Rico Water Resources Authority data, so we could not corroborate results independently. If MAP from these publications is correct, and runoff measured at the current gaging station is accurate, the Icacos watershed would fall along the tropical forest trend line (Fig 6).

We estimate similar MAP in the Bisley watersheds as previous studies (Table 1); if the relation between rainfall and runoff were similar to other tropical forest watersheds [27] and nearby Luquillo watersheds (Fig 6), runoff in the Bisley 2 watershed should be ~2,080 mm y^-1^ (Fig 6). However, previous studies have reported mean annual runoff estimates of 1,267–1,828 mm y^-1^, or 12–40% lower [45, 93, 100]. The Bisley watersheds are small and steep and thus difficult to measure accurately. Stream discharge in these watersheds has been estimated by measuring stage with a pressure transducer and converting to discharge using a stage-discharge relation [31, 101]. These watersheds are not gaged by the USGS and we did not examine the accuracy of their records. If runoff has been underestimated in the Bisley 2 watershed, water-budget derived ET and subsequent estimates of canopy interception [31] cited in global assessments as being “exceptionally high” and poorly simulated by existing models [36, 102], may not be valid.

Cloud-water interception is not recorded by standard rain gages and may contribute to MAP at elevations above the lifting condensation level (LCL) in the watersheds evaluated here. Clouds are present on the windward side of Cuchilla el Duque at elevations above 900 m during 80% of nighttime hours and 49% of daytime hours, respectively [105]. However, only 2–4% of the Mameyes and Fajardo watersheds, and none of the Sabana or Bisley subwatersheds, are above 900 m. Cloud-water contribution to the water budget is likely to be less in leeward watersheds than windward watersheds of the Luquillo Mountains due to a smaller area above 900 m (0.4–1% in the Gurabo and Canóvanas watersheds); it is also possible that after losing moisture to the windward slopes, air is less humid and would require lower temperatures, and thus higher elevations, to condense. The altitude of the LCL in the Luquillo Mountains, based on calculations from mean temperature and dew point, was reported to be higher on the northwestern slopes than on the southeastern slopes of the Luquillo Mountains [10]; this contrasts with a more recent spatial model [17].

### Possible mechanisms for spatial patterns of rainfall in the Luquillo Mountains

**Fig 7:**
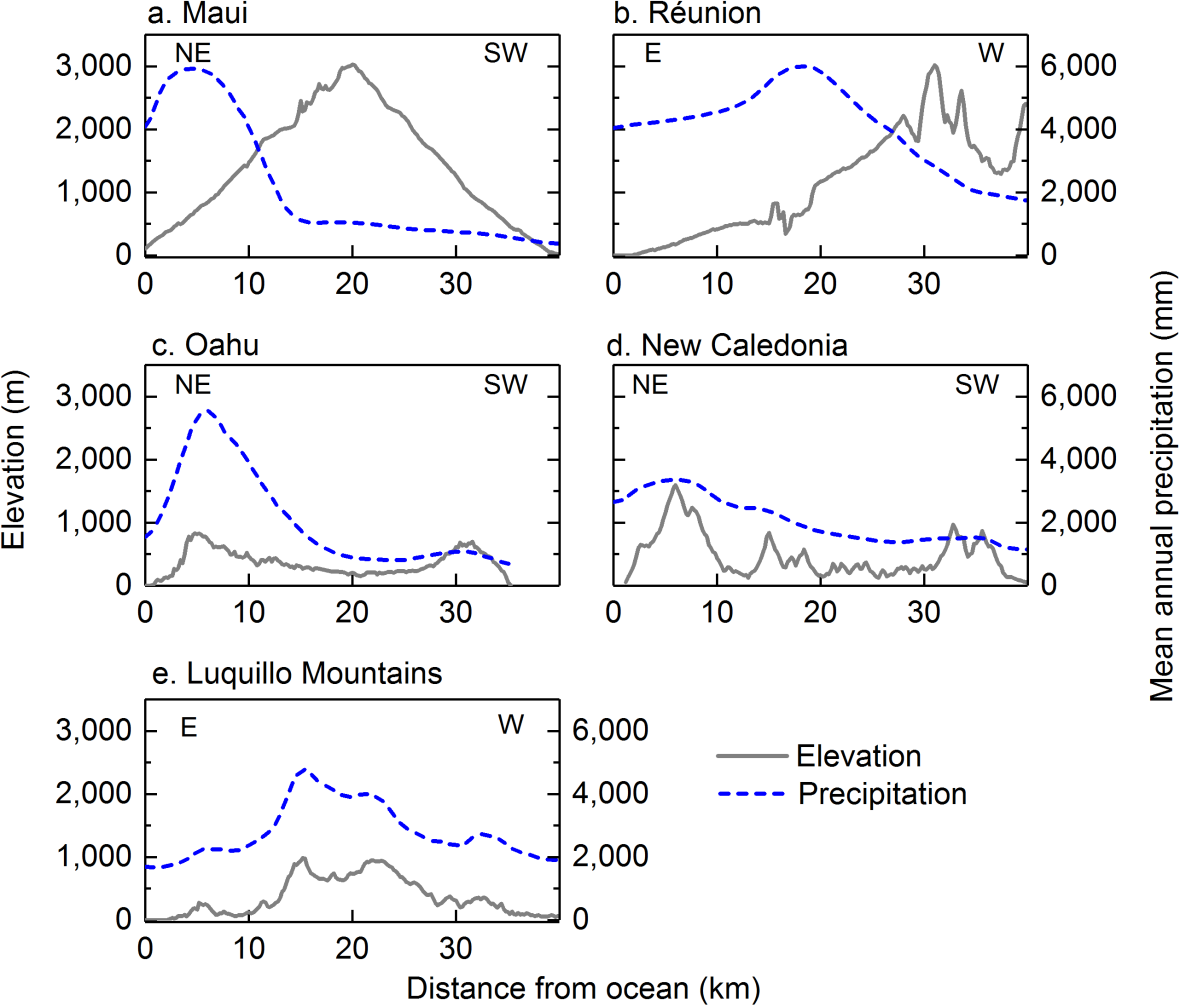
Elevation and mean annual precipitation versus distance from the ocean from dominant wind direction for islands within the trade-wind region: a) Maui; b) Réunion; c) Oahu; d) New Caledonia; e) Luquillo Mountains, Puerto Rico (spatial model of Fig 5A).

This analysis demonstrates that the Luquillo Mountains, like other mountain ranges with a strong prevailing wind direction, receive more precipitation on windward slopes than on leeward slopes. The difference between precipitation amounts delivered to windward and leeward slopes of mountain ranges, and the location of maximum precipitation relative to the peaks, varies depending on the height and width of the mountain range [106]. When tropical island mountains extend above the trade-wind inversion, as on the islands of Maui and Réunion, maximum MAP occurs windward of the peak, and leeward slopes receive substantially less MAP than windward slopes at equivalent elevations (Fig 7A and 7B). When mountains are narrower and shorter, and below the trade-wind inversion, as on the islands of Oahu and New Caledonia, maximum MAP typically occurs at or near the peak (Fig 7C and 7D); windward slopes receive more MAP than leeward slopes, but the difference is not as large. The highest peaks in the Luquillo Mountains, at 1,050–1,075 m in elevation, are below the average altitude of the cloud-capping trade-wind inversion (2,140 m in eastern Puerto Rico, based on San Juan radiosonde data [107]. A transect of elevation and MAP (estimated by our spatial model) through the Luquillo Mountains from the east shows that maximum MAP is located near the first major orographic barrier (Fig 7E), similar to Oahu and New Caledonia, and also to the relatively low mountain range of Dominica, another Caribbean island (not shown) [108].

**Fig 8:**
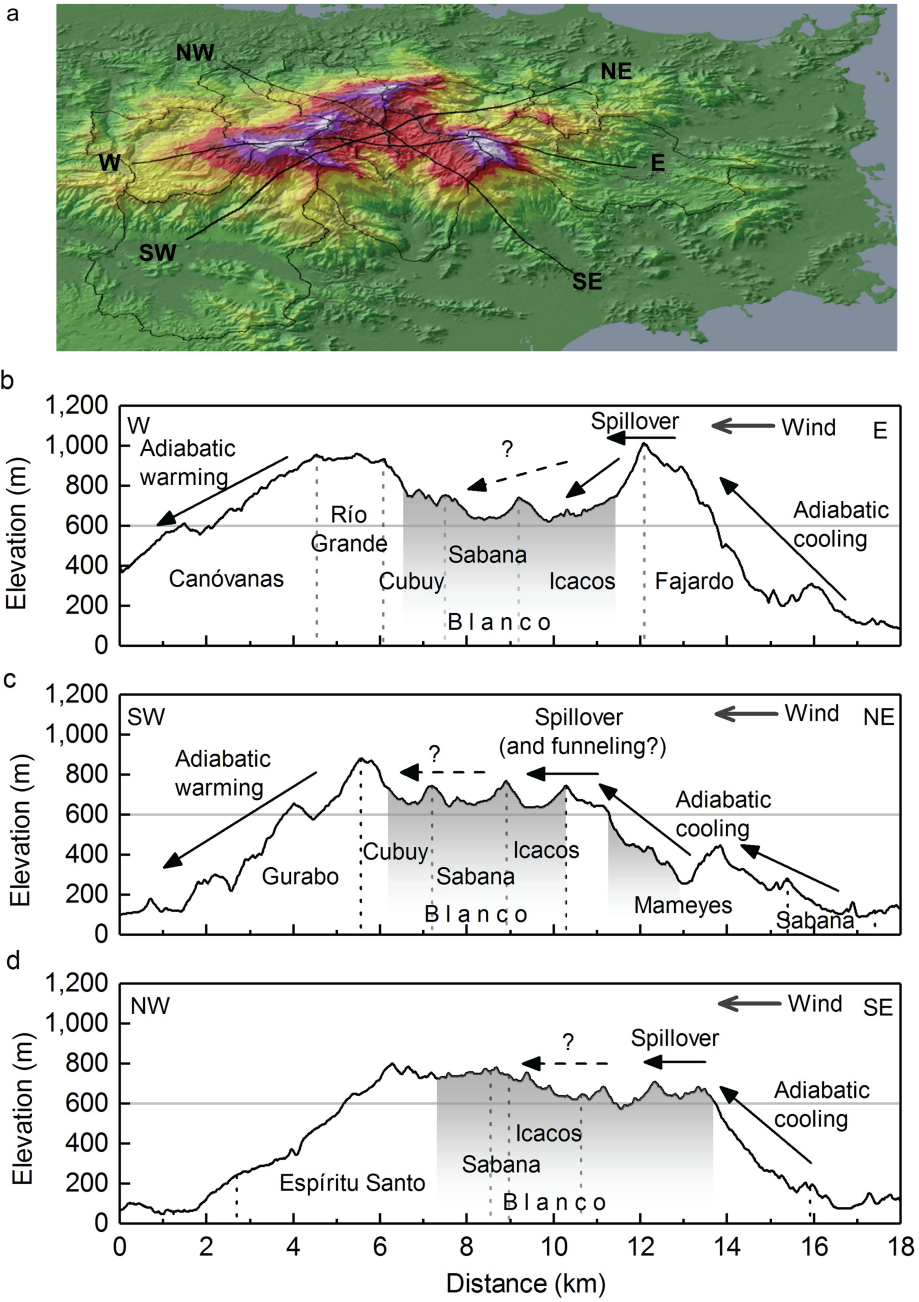
a) Three-dimensional view of elevation and topography of Luquillo Mountains showing a) potential path of dominant wind directions and watershed outlines, and b-d) cross-sections of elevation [78], watershed boundaries (dashed vertical lines), location of granodiorite intrusion [117] (gray shading), 600-m elevation (horizontal gray line) and potential precipitation mechanisms.

If lower mountain ranges have a narrow ridge, a high wind speed, and/or a long transit time for condensed water, precipitation can actually be enhanced on the lee side [109]. Maximum precipitation on the island of Oahu, for example, occurs about one kilometer leeward of the peak (Fig 7C). Precipitation that passes over an orographic barrier has been termed by some as “spillover” (e.g., [110–112]). The spatial model presented here suggests that this may also occur in the Luquillo Mountains (Figs 5A and 7E). Indeed, two rain gages on the western, downwind flank of Cuchilla el Duque (gages 37 and 42 in Fig 2) had substantially higher rainfall than similar-elevation gages from the same studies on the eastern flank (30 and 6, respectively), suggesting that MAP may be higher to the immediate lee of the ridge than on the windward side [23]. Several mechanisms may lead to this phenomenon. When the elevation of a hill or small mountain is low enough that a passing cloud is advected over the hilltop and not fully evaporated by downslope flow, precipitation can be carried to the lee side [106]. In landscapes with persistent fog, as is common at higher elevations along Cuchilla el Duque (such as Pico del Este and Pico del Oeste; Fig 1), a shallow orographic cloud can form in the low-level upslope flow, while a higher-level cloud can be advected over the peak; precipitation particles from the upper cloud grow by accretion of cloud water in the lower cloud, and precipitation falls on both windward and leeward slopes (this is known as a “seeder-feeder” mechanism [113, 114]). In addition, hills or small mountains can generate lee waves that may lead to convective clouds; if the terrain in the lee of the hill has a general upward slope, the lee wave may lead to leeward rain bands parallel to the flow over the hill [106]. These mechanisms may lead to high precipitation in the Icacos watershed when winds are from the east, the most common direction (Fig 8A and 8B). However, frequent winds from the northeast [69, 70] reaching Cuchilla el Duque can pass through a 750-m high gap in the ridge (Fig 8A and 8C); winds may accelerate through the gap [115] and transport precipitation further downwind. When winds are from the southeast (about 14% of the year [70]), moist, ocean-derived air could rise up the Blanco/Icacos valley with no orographic barrier to entry to the Icacos watershed (Fig 8D). In this case, simple adiabatic cooling could cause high precipitation in that watershed. Finally, thermal instability centered over the mountains produces convection (thunderstorms) with daytime heating, especially during periods with a weaker trade-wind inversion [33, 116]. The dominant mechanism(s) delivering precipitation to the Icacos watershed will vary throughout the year, when different storm systems, originating from different directions, affect the area.

While many possible scenarios may contribute to high MAP in the Icacos watershed, it is difficult to speculate how these scenarios affect the further leeward, western Blanco (Sabana and Cubuy subwatersheds), due to lack of rain gages (Fig 2) and/or other meteorological data. Much of the upper Blanco watershed is on a (relative) plateau. With elevations varying only between 650–800 m in a ~5–8 km distance (Figs 1 and 8), air moving over this plateau does not undergo downslope flow. On Cuchilla el Duque, and in the Icacos watershed (within 2 km west of the ridge), MAP is enhanced by a factor of 7 compared to MAP over the ocean immediately to the east of Puerto Rico (~730 mm y^-1^ [118]). The rainfall enhancement factor decreased to 2.7–4.1 at gages in the Gurabo and Canóvanas watersheds (at elevations 340–790 m). These decreases are similar to those observed in the Southern Alps of New Zealand, where MAP decreased only slightly from 5,000 mm at a 918-m ridge (an enhancement factor of ~4.8, based on average ocean MAP at that latitude [119] to 4,500 mm at 750 m (6.5 km leeward), but decreased to 2,100 mm (enhancement factor of ~2.0) 8 km further leeward at 639 m elevation [120]. During a prolonged northwesterly storm in the Southern New Zealand Alps, the percent of total transect precipitation falling in leeside catchments varied between 11–70%, and was highest during stronger flows and reduced static stability [112]; “spillover” reached a distance between 6–29 km from the barrier. Due to lack of gages and other meteorological data in much of the Blanco watershed, we cannot currently estimate the extent of spillover there.

Smaller topographic features in eastern Puerto Rico, such as the 250-650-m ridge that separates the Sabana watershed from the Mameyes and Fajardo watersheds (Figs 1 and 8C), may also affect spatial variability. Two gages on the lower, western slope of the ridge between the Mameyes and Sabana watersheds (gages 3 and 4 in Fig 2) had >10% more MAP than predicted by PRISM [6], even with one being located on a tower, which often results in underreported MAP. Some of the enhanced leeside rainfall mechanisms described earlier may be relevant here. Also, greater precipitation on the leeward side of relatively low (200 m) hills may sometimes be caused by redistribution of precipitation as a result of altered raindrop trajectories by wind [121]. Average wind speeds at a tower on the ridge were 4 m s^-1^ compared to 1–2 m s^-1^ at a lower tower (gage 4 in Fig 2) in the Bisley watershed [122]. Thus, wind speeds likely play a critical role in spatial distribution of precipitation.

### Suggested research directions

We have identified gaps in both spatial and mechanistic understanding of precipitation in the Luquillo Mountains. Strategically placed rain gages—such as on both sides of orographic barriers, particularly in areas where no rain gages have ever been installed—would improve our spatial understanding of precipitation patterns. The physical setting of such rain gages should be chosen to minimize wind-related effects and vegetation blocking of either direct or angled precipitation. In order to account for the effects of wind, well-maintained, shielded rain gages, and measurements of wind speed, wind direction, and temperature would be needed, as would corrections based on wind losses, inclined rainfall, and the effects of slope [26, 122]. A comparison of rain gage types, to ensure that both low-intensity and high-intensity rainfall are accounted for (e.g., [89]), is warranted. Evaluation of sub-daily rainfall rates would be required to determine whether the degree of diurnal cycling is greater on leeward slopes, indicating a greater role of surface heating than convection forced by orographic uplift, and whether leeward watersheds benefit from trade wind-derived orographic precipitation. Unfortunately, the region has many issues that make accurate precipitation measurement challenging—steep and uneven surfaces, ground covered by a continuous expanse of forest, rapidly growing vegetation, limited accessibility, and precipitation that sometimes falls as a fine mist and other times as high-intensity rainfall. Other studies that have identified mechanisms and effects of precipitation variability at the scale of the Luquillo Mountains have incorporated indirect methods of estimating rainfall, such as ground-based Doppler radar, research aircraft, atmospheric observations, and/or intensive modeling (e.g., [108, 112, 123]), which could fill in spatial gaps (but are costly). Calibration of radar with the extensive dataset provided here is a potential avenue for improving estimates of precipitation in the Luquillo Mountains.

Further studies of the amount of precipitation delivered to different regions of the Luquillo Mountains by various weather systems (e.g., [67]) are required if we are to understand the importance of different storm types, and seasonality, on water delivery in the region. Synoptic-scale storms affecting eastern Puerto Rico can have widely different wind directions and speeds, moisture content, and frontal stability. Studies in other regions have shown that the watershed in which flooding occurs can be determined by slight (10 degree) differences in wind direction [124]. The tangential winds embedded in hurricanes can produce greatly enhanced upslope flow, and depending on the height of the mountain, may produce maximum rainfall on either the windward or leeward side [106, 123]. Improved understanding of spatial and temporal variability of rainfall will be critical if hurricane strength increases in the future, as is projected [125].

A more extensive accounting of water diversion amounts and timing (e.g., [126, 127]) in the Luquillo Mountains is needed to develop accurate water budgets and improve our understanding of the relation between precipitation and runoff (Fig 6). Field measurements of ET, and comparison to water-balance-derived ET, would enhance our understanding of ecohydrological processes. This would also provide additional information for the debate on whether hydrological processes differ in maritime and continental tropical forests [39, 40, 122]. The lack of rainfall data in the Luquillo Mountains has hampered the ability to assess potential effects of climate change in eastern Puerto Rico [56]; climate-change response cannot be accurately assessed when *current* rates of MAP and ET are not yet understood.

We hope this work will lead to the improvement of hydrologic models, ecohydrological interpretations, and assessments of climate change effects in the Luquillo Mountains. The results of this study also have implications for other areas of research. As one example, the role of geologic composition and tectonics in the geomorphology of the region has been an important topic of research for decades (e.g., [128–131]. Yet spatial variability in precipitation amount and intensity may play a role in sculpting this mountain range (Fig 8); we hope that the new paradigm of spatial variability in precipitation provides a new parameter to consider in the ongoing discussion of erosion rates in the Luquillo Mountains. Other relevant topics are vegetation distribution, the role of vegetation types and interception rates, sediment erosion and transport processes, wetland delineation, and stream chemistry.

## Supporting information

S1 Table Locations, type of gage, periods of record, and other details for rain gages in the Luquillo Mountains, Puerto Rico. (XLSX)

S1 Fig Maps of elevation in the Luquillo Mountains filtered to a various diameters and scatterplots showing the relation between those wavelengths and measured mean annual precipitation. (PDF)

S2 Fig Maps of Luquillo Mountains, Puerto Rico, showing mean annual precipitation as predicted by spatial model developed from 1-km wavelength/MAP regression and residual interpolation (high-resolution pdf with geospatial data included; Puerto Rico UTM Zone 20N). (PDF)
