# Supplementary material for: Reassessing rainfall in the Luquillo Mountains, Puerto Rico: Local and global ecohydrological implications
Source: PLoS One. 2017 Jul 7;12(7):e0180987. doi: 10.1371/journal.pone.0180987 (PMC5501619; doi:10.1371/journal.pone.0180987)
Supplement: S1 Fig — (PDF) [file pone.0180987.s002.pdf]

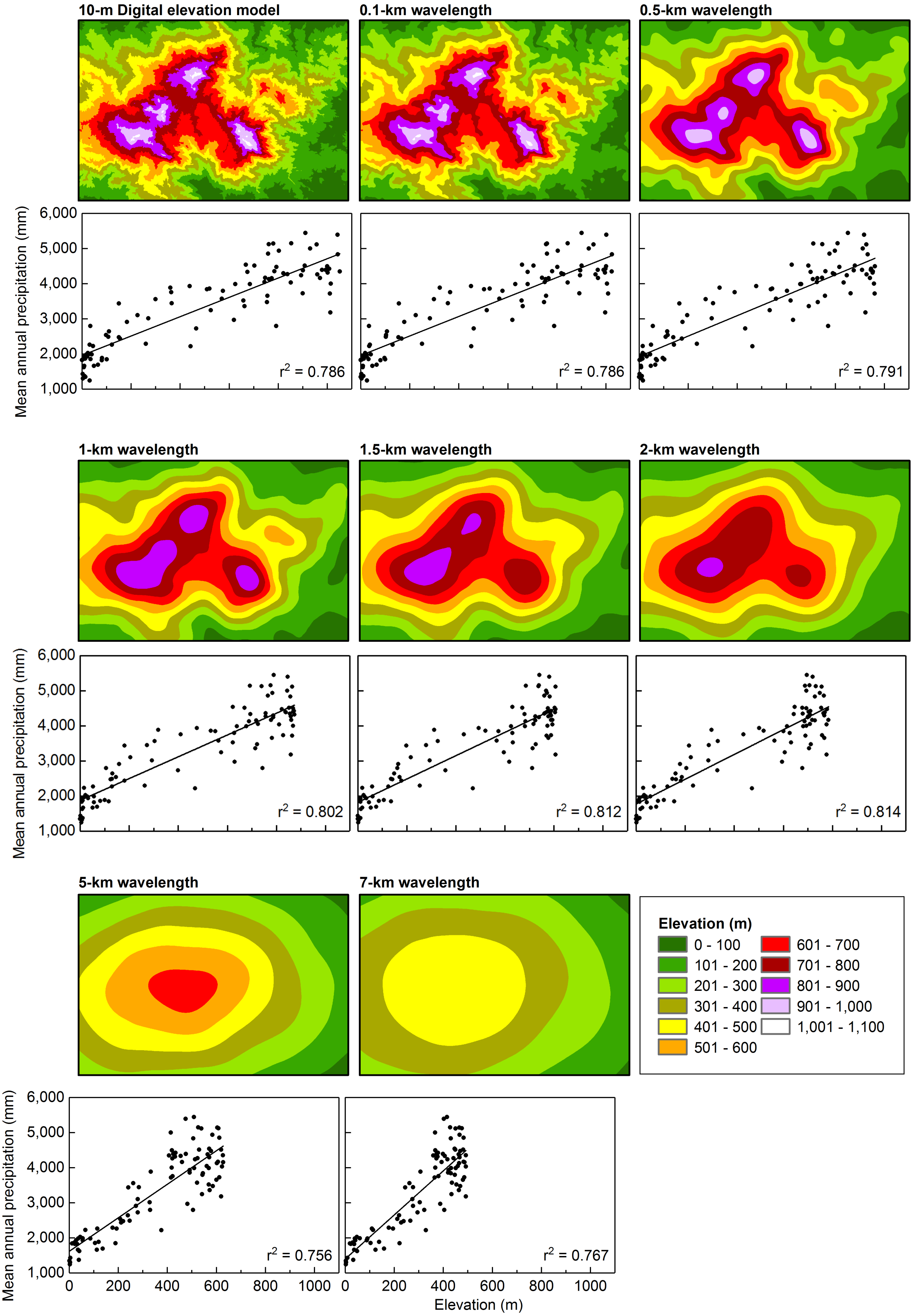

S1 Figure. Maps of elevation in the Luquillo Mountains, Puerto Rico, filtered to various diameters, and scatterplots showing the relation between those wavelengths and measured mean annual precipitation.
