# Supplementary figures and images for: Reassessing rainfall in the Luquillo Mountains, Puerto Rico: Local and global ecohydrological implications
Source: PLoS One. 2017 Jul 7;12(7):e0180987. doi: 10.1371/journal.pone.0180987 (PMC5501619; doi:10.1371/journal.pone.0180987)

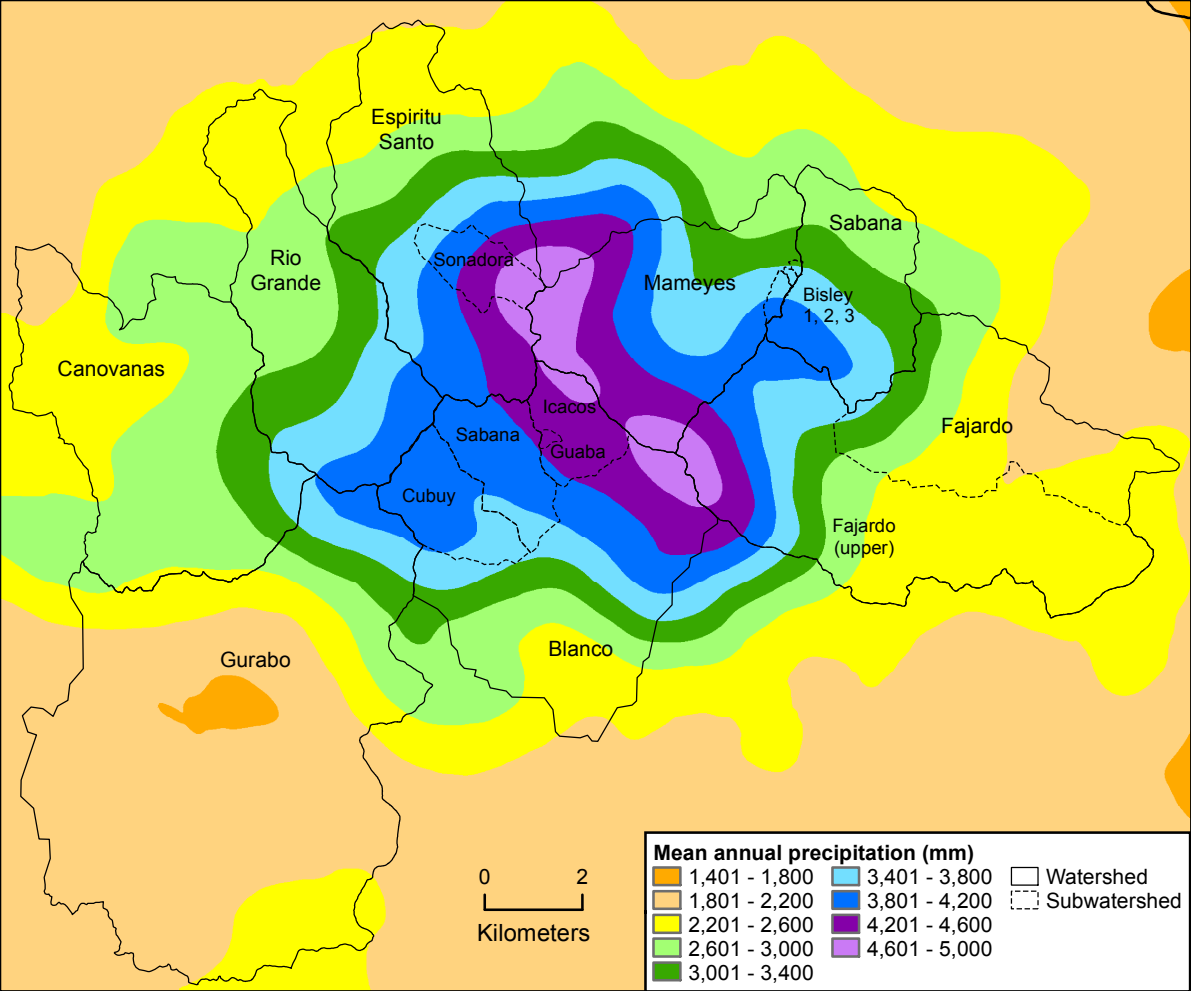

Supplement: S2 Fig — (PDF) [file pone.0180987.s003.pdf]
